# Draft genome sequence of carbapenem-resistant *Klebsiella pneumoniae* ST6260 isolated from the catheter tip of a female patient in Nepal

**DOI:** 10.1128/mra.00145-25

**Published:** 2025-05-27

**Authors:** Suchitra Thapa, Basudha Shrestha, Dev Raj Joshi, Reshma Tuladhar, Maria Getino, Manisha Shrestha, Yojana Pokhrel, Elita Jauneikaite

**Affiliations:** 1 Department of Microbiology, Tribhuvan University 97983, Kirtipur, Central Development Region, Nepal; 2 Department of Microbiology, Kathmandu Model Hospital, Kathmandu, Nepal; 3 Central Department of Microbiology, Tribhuvan University 282612, Kirtipur, Nepal; 4 NIHR Health Protection Research Unit in Healthcare Associated Infections and Antimicrobial Resistance, Department of Infectious Disease, Imperial College London 4615, London, United Kingdom; Wellesley College, Wellesley, Massachusetts, USA

**Keywords:** antibiotic resistance, *Klebsiella*

## Abstract

*Klebsiella pneumoniae* is an opportunistic human pathogen, particularly associated with nosocomial infections and multidrug resistance. Here, we present a draft genome sequence of a carbapenem-resistant *K. pneumoniae* ST6260 isolated from the catheter tip of a female patient in a referral case received at Kathmandu Model Hospital, Kathmandu, Nepal.

## ANNOUNCEMENT

*Klebsiella pneumoniae* was isolated from the catheter tip of a 27-year-old female. A 5 cm distal tip was received in a sterile container at the hospital (N27°42′9.1656″, E85°19′12.5832″), cultured on MacConkey agar (HiMedia, India) plate using the rolled plate method (1), and then incubated at 37°C for 24 hours. A single colony from the plate was grown in Nutrient broth (HiMedia, India) at 37°C for 4 hours and turbidity matched with 0.5 McFarland for antibiotic susceptibility testing. Antibiotic susceptibility was detected by the disc diffusion method, and carbapenemase production was confirmed by the modified carbapenem inactivation method, as described by CLSI guidelines (2). The isolate was preserved in Tryptic Soy Broth (HiMedia, India) (with 20% glycerol) at −80°C. Later, it was revived onto a Nutrient agar (HiMedia, India) plate for DNA extraction. A single colony from the plate was transferred into Luria-Bertani (LB) (HiMedia, India) broth for overnight incubation at 37°C. The DNA was extracted from LB broth using the CTAB method (3), and quality was determined through 260/280 nm absorbance measures using a NanoDrop spectrophotometer 1000 (Thermo Scientific, USA). Then, the genomic DNA was sent to MicrobesNG for whole-genome sequencing.

Genomic DNA libraries were prepared using the Nextera XT Library Prep Kit (Illumina, USA) following the manufacturer’s protocol with the following modifications: input DNA was increased twofold, and PCR elongation time was increased to 45 seconds. DNA quantification and library preparation were carried out on a Hamilton Microlab STAR automated liquid handling system (Hamilton Bonaduz AG, Switzerland). Libraries were sequenced on an Illumina NovaSeq 6000 (Illumina, USA) using a 250 bp paired end protocol (~890,000 reads per pair, average coverage 70×). Raw reads were checked using FastQC version 0.11.9 (4). Trimmomatic version 0.39 (5) was used to trim reads with the following parameters: LEADING:20 TRAILING:20 SLIDING WINDOW:5:30 MINLEN:50. Species identification was done with Kraken2 version 2.1.43 (6) and Bracken version 2.9 (7). *De novo* assembly was performed using SPAdes version 3.15.2 (8) with careful mode activated and selecting k-mers up to 127. Bioawk version 1.0 (9) was then used to filter out contigs with size <200 bp and SPAdes coverage <2. Assembly statistics were assessed using QUAST version 5.0.2 (10) and CheckM2 version 1.2.3 (11). PGAP version 6.9 (12) was used for annotation. Kleborate version 2.5 (13) was used to report MLST (13) (BIGSdb database), virulence loci, AMR genes, and K and O locus via Kaptive version 2.0.4 (14). Plasmid reconstruction was performed using MOB-suite version 3.1.8 (15). Default parameters were used except where otherwise stated.

Draft genome assembly stats were as follows: 5,568,701 bp, 117 contigs, 57% GC content, and *N*_50_ of 187,282 bp. The draft genome contains a total of 5,585 genes, of which 5,321 were coding DNA sequences, 79 tRNA, 27 rRNA (6 complete and 21 partial), and 149 pseudogenes. *K. pneumoniae* genotype was ST6260. The predicted capsule type was K17 (locus K17) and O type O1 (locus O1/O2v1). Kleborate predicted a virulence score of 1 with yersiniabactin *ybt9* and truncated ICEKp3, and a resistance score of 2, indicating that carbapenemase without colistin resistance was detected, regardless of ESBL genes or OmpK mutations. AMR determinants and plasmids identified are summarized in Table 1.

**TABLE 1:** Summary of AMR determinants identified in the *K. pneumoniae* ST6260 genome from Nepal^*a*^

	Size (bp)	No. of contigs	AMR determinants
Chromosome	5,318,348	91	bla*SHV-1*, *dfrA12*, *aadA2*, *fosA*, *oqxA*, *oqxB;* GyrA-83Y, GyrA-87G, ParC-80I
Plasmid 1 (IncFIB, IncFII, IncR; mobilizable)	148,151	18	bla*TEM-1A*, bla*OXA-9*, *ant(3″)-Ia*, *aac(6')-Ib*, *sul1*, bla*NDM-5*, *erm(B*), *mph(A*), *rmtB*
Plasmid 2 (IncFIB; non-mobilizable)	43,101	2	–^*b*^
Plasmid 3 (no rep; mobilizable)	23,345	2	–
Plasmid 4 (no rep; non-mobilizable)	19,072	1	–

^*a*^ In total, seven plasmids were reported by MOB-suite analysis; only plasmids larger than 10 kbp have been summarized in the table.

^*b*^ “–” indicates not found.

## Data Availability

The raw reads for *K. pneumoniae* isolate are submitted to European Nucleotide Archive under sample accession number SAMEA115926202, https://www.ebi.ac.uk/ena/browser/view/SAMEA115926202, while draft assembly has been deposited in GenBank under the BioProject accession number PRJEB78979, https://www.ncbi.nlm.nih.gov/bioproject/1155534.

## References

[1] Cheesbrough M. 2010. District laboratory practice in tropical countries. Part 2. 2nd ed. Cambridge University Press.

[2] Clinical and Laboratory Standards Institute. 2023. In Performance standards for antimicrobial susceptibility testing, 33rd ed. CLSI, Wayne, PA, USA.

[3] Jaufeerally-Fakim Y, Dookun A. 2000. Extraction of high quality DNA from polysaccharides secreting Xanthomonads. University of Mauritius Research Journal 6:www.ajol.info/index.php/umrj/article/view/130838.

[4] Andrews S. 2010. FastQC: A Quality Control Tool for High Throughput Sequence Data. Available from: www.bioinformatics.babraham.ac.uk/projects/fastqc/

[5] Bolger AM, Lohse M, Usadel B. 2014. Trimmomatic: a flexible trimmer for Illumina sequence data. Bioinformatics 30:2114–2120. doi:10.1093/bioinformatics/btu170 24695404 PMC4103590

[6] Wood DE, Lu J, Langmead B. 2019. Improved metagenomic analysis with Kraken 2. Genome Biol 20:257. doi:10.1186/s13059-019-1891-0 31779668 PMC6883579

[7] Lu J, Breitwieser FP, Thielen P, Salzberg SL. 2017. Bracken: estimating species abundance in metagenomics data. PeerJ Comput Sci 3:e104. doi:10.7717/peerj-cs.104 PMC12016282 40271438

[8] Bankevich A, Nurk S, Antipov D, Gurevich AA, Dvorkin M, Kulikov AS, Lesin VM, Nikolenko SI, Pham S, Prjibelski AD, Pyshkin AV, Sirotkin AV, Vyahhi N, Tesler G, Alekseyev MA, Pevzner PA. 2012. SPAdes: a new genome assembly algorithm and its applications to single-cell sequencing. J Comput Biol 19:455–477. doi:10.1089/cmb.2012.0021 22506599 PMC3342519

[9] Li H. 2018. Bioawk (v1.0). Available from: https://github.com/lh3/bioawk

[10] Gurevich A, Saveliev V, Vyahhi N, Tesler G. 2013. QUAST: quality assessment tool for genome assemblies. Bioinformatics 29:1072–1075. doi:10.1093/bioinformatics/btt086 23422339 PMC3624806

[11] Chklovski A, Parks DH, Woodcroft BJ, Tyson GW. 2023. CheckM2: a rapid, scalable and accurate tool for assessing microbial genome quality using machine learning. Nat Methods 20:1203–1212. doi:10.1038/s41592-023-01940-w 37500759

[12] Tatusova T, DiCuccio M, Badretdin A, Chetvernin V, Nawrocki EP, Zaslavsky L, Lomsadze A, Pruitt KD, Borodovsky M, Ostell J. 2016. NCBI prokaryotic genome annotation pipeline. Nucleic Acids Res 44:6614–6624. doi:10.1093/nar/gkw569 27342282 PMC5001611

[13] Lam MMC, Wick RR, Watts SC, Cerdeira LT, Wyres KL, Holt KE. 2021. A genomic surveillance framework and genotyping tool for Klebsiella pneumoniae and its related species complex. Nat Commun 12:4188. doi:10.1038/s41467-021-24448-3 34234121 PMC8263825

[14] Wyres KL, Wick RR, Gorrie C, Jenney A, Follador R, Thomson NR, Holt KE. 2016. Identification of Klebsiella capsule synthesis loci from whole genome data. Microb Genom 2:e000102. doi:10.1099/mgen.0.000102 28348840 PMC5359410

[15] Robertson J, Nash JHE. 2018. MOB-suite: software tools for clustering, reconstruction and typing of plasmids from draft assemblies. Microb Genom 4:e000206. doi:10.1099/mgen.0.000206 30052170 PMC6159552

