# Erratum for Thapa et al., “Draft genome sequence of carbapenem-resistant *Klebsiella pneumoniae* ST6260 isolated from the catheter tip of a female patient in Nepal”

**DOI:** 10.1128/mra.00585-25

**Published:** 2025-08-15

**Authors:** Suchitra Thapa, Basudha Shrestha, Dev Raj Joshi, Reshma Tuladhar, Maria Getino, Manisha Shrestha, Yojana Pokhrel, Elita Jauneikaite

## ERRATUM

Volume 14, Issue 6, e00145-25, 2025, https://doi.org/10.1128/mra.00145-25. Page 2, Author Affiliations: Suchitra Thapa’s affiliation should read “Department of Microbiology, Amrit Campus, Tribhuvan University, Kathmandu, Nepal.”

